# Robot-assisted versus navigation-assisted screw placement in spinal vertebrae

**DOI:** 10.1007/s00264-022-05638-0

**Published:** 2022-11-24

**Authors:** Tong Yu, Jian-Hang Jiao, Yang Wang, Qing-Yu Wang, Wei-Bo Jiang, Zhong-Han Wang, Min-Fei Wu

**Affiliations:** Department of Orthopedic Medical Center, The Second Hospital of Jilin University, Changchun, Jilin Province China

**Keywords:** Robot, Navigation, Spine, Screw placement

## Abstract

**Purpose:**

Both robots and navigation are effective strategies for optimizing screw placement, as compared to freehand placement. However, few studies have compared the accuracy and efficiency of these two techniques. Thus, the purpose of this study is to compare the accuracy and efficiency of robotic and navigation-assisted screw placement in the spinal vertebrae.

**Methods:**

The 24 spine models were divided into a robot- and navigation-assisted groups according to the left and right sides of the pedicle. The C-arm transmits image data simultaneously to the robot and navigates using only one scan. After screw placement, the accuracy of the two techniques were compared using “angular deviation” and “Gertzbein and Robbins scale” in different segments (C1–7, T1–4, T5–8, T9–12, and L1–S1). In addition, operation times were compared between robot- and navigation-assisted groups.

**Results:**

Robots and navigation systems can simultaneously assist in screw placement. The robot-assisted group had significantly less angular deviation than the navigation-assisted group from C1 to S1 (*p* < 0.001). At the C1–7 and T1–4 segments, the robot-assisted group had a higher rate of acceptable screws than the robot-assisted group. However, at the T5–8, T9–12, and L1–S1 segments, no significant difference was found in the incidence of acceptable screws between the two groups. Moreover, robot-assisted screw placement required less operative time than navigation (*p* < 0.05).

**Conclusion:**

The robot is more accurate and efficient than navigation in aiding screw placement. In addition, robots and navigation can be combined without increasing the number of fluoroscopic views.

**Supplementary Information:**

The online version contains supplementary material available at 10.1007/s00264-022-05638-0.

## Introduction

The pedicle screw technique is an important technique in the field of spinal surgery [1]. Conventional freehand pedicle screw implantation requires an experienced surgeon to ensure screw accuracy and is associated with high surgical risk [1]. Due to its proximity to the spinal canal and surrounding vessels, misplacement of the pedicle screw can result in injury to adjacent nerve structures and vessels, eventually causing neurovascular complications [2–4].

In 1995, Amiot [5] first described pedicle screw fixation using a computer navigation system, and in the following years, this technology has dramatically developed [6]. With the advantages of real-time intra-operative images and three-dimensional navigation, navigation is considered a useful technology for improving surgical accuracy and reducing surgical complications. Moreover, in 2016, Tian first reported a robotic surgical procedure using the Tianji robot to improve the accuracy of implanted screw positioning [7]. The Tianji robot is a multi-indication orthopaedic robot approved for clinical use by the China Food and Drug Administration. With pre-operative planning and real-time robotic arm guidance, robotic technology has been reported to improve screw insertion precision during spine surgery [7–10]. Therefore, both robots and navigation are scientific and technical means to guide pedicle screw placement and improve surgical safety.

However, to the best of our knowledge, few studies have compared the accuracy and efficiency of these two techniques. Therefore, this study investigated the accuracy and efficiency of robot-assisted versus navigation-assisted pedicle screw implantation in spinal models.

## Materials and methods

### Equipment information

**Fig. 1:**
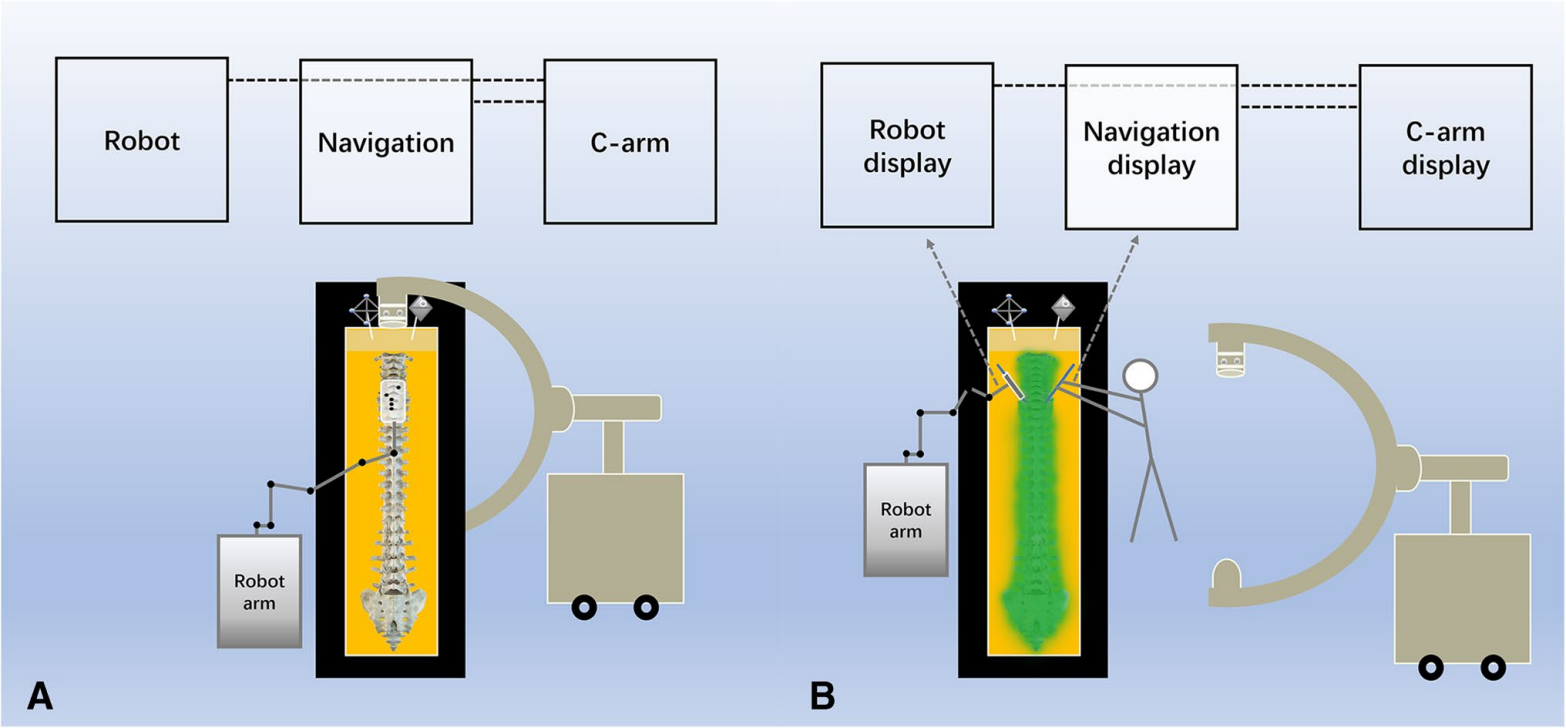
Position of the robot, navigation, C-arm, and Jackson surgical table in the operating room (**A**). Robotics and navigation simultaneously guide screw placement in the bilateral pedicle (**B**)

The robot-assisted system (TINAVI Medical Technologies, Ltd., China) consists of a robotic arm, optical tracking system, robotic workstation, and a navigation toolkit. The navigation-assisted system consists of a workstation with navigation software (The Stryker Spine Navigation System, USA) and specific surgical instruments. The C-arm type is the Arcadis Orbic 3D (Siemens, Medical Solutions, Erlangen, Germany) (Fig. 1A) (Supplemental information 1).

### Model preparation

Twenty-four simulation spine models (Sawbone, Pacific Research Laboratories, Inc.) made up of high-resolution foam cortical shells with cancelli were all radiolucent to optimize image quality and minimize artifacts. The 24 spine models were divided into two groups according to the left and right sides of the pedicle (left: robot-assisted group; right: navigation-assisted group). Each spine model was embedded in a sponge by an assistant, and the bilateral transverse processes were completely covered by colored sand, exposing only the same operative field as that in clinical practice (Fig. 1B). The spine model was placed in a rectangular slot surrounded by a transparent acrylic plate, which was installed with the tracers of the robot and navigation system (Fig. 1B).

### Robot-assisted screw placement

Image acquisition of the spinal vertebrae was performed using a C-arm. The image data were transferred to the robot workstation via a network cable. Screw planning, including diameter, length, and trajectory, was carried out in the robotic workstation. First, the sleeve was placed into the screw guide. Then, after the foot pedal was pressed, the robot arm automatically adjusted its direction according to the trajectory planned for the surgery until it exactly matched the planned trajectory. Next, K-wire was drilled into the vertebrae. The cannulated tap was used to prepare the screw channel first; then, the pedicle screw was implanted (Fig. 1B) (Supplemental information 2).

### Navigation-assisted screw placement

The SpineMap 3D 2.0 was selected in the navigation workstation. The instrument tracker, patient tracker, and C-arm tracker were activated. The navigation system and C-arm were connected using a network cable. The image data from the C-arm, which was transmitted to the robot workstation, was sent to the navigation workstation. Next, a screw design was performed. The screw view model of navigation, which is an accurate and easy-to-use model, was selected in the assisted screw placement mode to aid the surgeon in establishing the screw channel with the tap in hand, followed by screw placement (Fig. 1B) (Supplemental information 2).

### Evaluation of outcomes

The accuracy and operative time of screw placement were compared between the two groups in different segments, including C1–7, T1–4, T5–8, T9–12, and L1–S1. After operation, the CT scans were performed on the spine models.

The accuracy of screw placement was assessed according to the angular deviation between the planned and actual screw positions. The angular deviation was defined as the angle between the central axes of the planned and the actual screw positions. Moreover, the accuracy of screw placement was evaluated using the Gertzbein and Robbins scale [11]. Grade A: screw is completely within the bone. Grade B: cortical breach is of less than 2 mm. Grade C: cortical breach is > 2 mm and less than 4 mm. Grade D: cortical breach is > 4 mm and less than 6 mm. Grade E: cortical breach is of more than or equal to 6 mm. The pedicle screw position was evaluated by a radiologist who was blinded to the study’s purpose. Good screw position was defined as grades A and B, whereas poor screw position was defined as grades C, D, and E.

The operative time for only one vertebra (C7) was recorded by an assistant, including the preparation time (robot/navigation boot time to C-arm scan), screw design time, and robot/navigation-guided screw placement time.

### Statistical analysis

To investigate whether a difference exists between the robot-assisted and navigation-assisted techniques at different segments of the spine, Fisher’s exact test and independent-sample *t*-test were used. Statistical significance was set at *p* < 0.05.

## Results

Robots and navigation systems can simultaneously assist in screw placement. A total of 1200 screws were inserted into the spine model from C1 to S1, including 600 screws in the robot group and 600 screws in the navigation group. In each group, the number of implanted screws was 168 in C1–7, 96 in T1–4, 96 in T5–8, 96 in T9–12, and 144 in L1–S1.

**Fig. 2:**
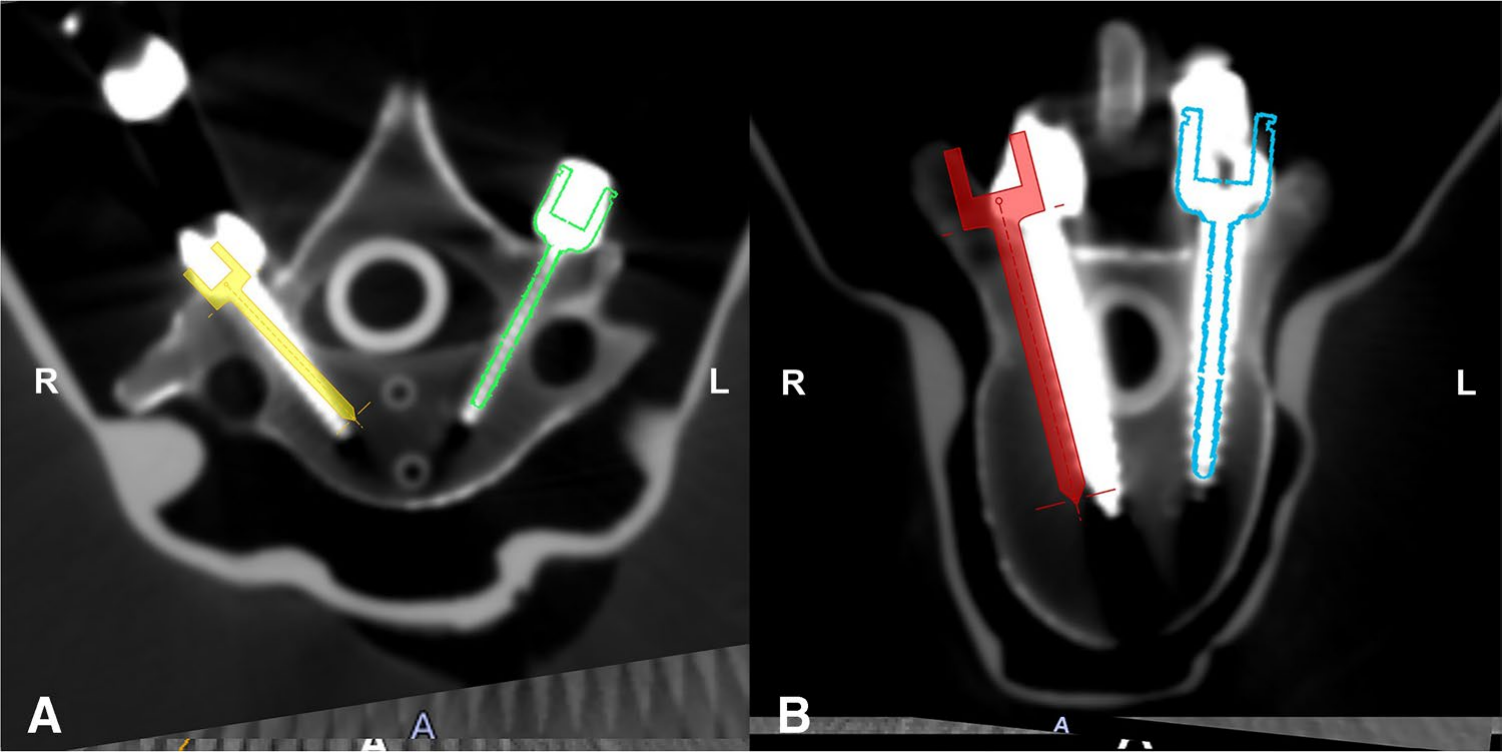
Angular deviation results for robot- and navigation-guided screw placement. **A** is the merged images of the C7 screw design and the placed screws, showing the angular deviation results of 0° and 8.7° for the robot and navigation guidance, respectively. **B** is the merged images of the T10 screw design and the placed screws. The Gertzbein and Robbins scale outcomes for robot- and navigation-guided screw placement were the grade A and grade D, respectively

Regarding the accuracy of screw placement, the angular deviation in the robot-assisted group was significantly less than that in the navigation-assisted group from C1 to S1 (*p* < 0.001) (Fig. 2) (Table 1). The Gertzbein and Robbins scale outcomes showed that at segments C1–7 and T1–4, the robot-assisted group had a higher rate of acceptable screws (grade A + B) than the robot-assisted group [97.02% vs. 91.67% (*p* < 0.05) and 98.96% vs. 91.67% (*p* < 0.05), respectively]. However, at the T5–8, T9–12, and L1–S1 segments, no significant difference was found in the incidence of acceptable screws between the robot-assisted group and navigation-assisted group [97.92% vs. 97.92% (*p* > 0.05), 100% vs. 98.96% (*p* > 0.05), 100% vs. 98.61% (*p* > 0.05), respectively] (Table 2).

**Table 1:** Comparison results of angular deviations between robot and navigation at different spinal segments

Segments	Robot group (angle deviations°)	Navigation group (angle deviations°)	*P* value
Cervical			
C1–7	1.938 ± 0.622	3.008 ± 0.736	< 0.001*
Thoracic			
T1–4	2.180 ± 0.466	2.912 ± 0.769	< 0.001*
T5–8	2.168 ± 0.469	3.983 ± 1.259	< 0.001*
T9–12	2.137 ± 0.306	4.854 ± 1.830	< 0.001*
Lumbosacral			
L1–S1	2.239 ± 0.455	4.901 ± 1.466	< 0.001*

The values are given using an independent sample *t*-test

^*^represents statistically significant differences

**Table 2:** Comparison results of Gertzbein and Robbins scale between robot and navigation at different spinal segments

Segments	Robot group grade A + B screws, no. (%)	Navigation group grade A + B screws, no. (%)	*P* value
Cervical			
C1–7	163/168 (97.02)	154/168 (91.67)	0.034*
Thoracic			
T1–4	95/96 (98.96)	88/96 (91.67)	0.041*
T5–8	94/96 (97.92)	94/96 (97.92)	1.000
T9–12	96/96 (100.00)	95/96 (98.96)	1.000
Lumbosacral			
L1–S1	144/144 (100)	142/144 (98.61)	1.000

The values are given using Pearson × ^2^ test

^*^represents statistically significant differences

For operation time, the screw-designed time showed no significant statistical difference between the robot and navigation-assisted groups [1.379 ± 0.265 vs. 1.425 ± 0.305 (*p* > 0.05)]. However, the preparation time and guided-screw insertion time in the robot-assisted group were significantly shorter than those in the navigation-assisted group [2.567 ± 0.253 vs. 3.758 ± 0.290 (*p* < 0.05), 2.800 ± 0.284 vs. 4.013 ± 0.464 (*p* < 0.05)] (Table 3).

**Table 3:** Comparison results of screw insertion time between robot and navigation at different spinal segments

Time	Robot group (min)	Navigation group (min)	*P* value
T1	2.567 ± 0.253	3.758 ± 0.290	< 0.001*
T2	1.379 ± 0.265	1.425 ± 0.305	0.582
T3	2.800 ± 0.284	4.013 ± 0.464	< 0.001*

T1 represents preparation time; T2 indicates screw-planed time; T3 is guided-screw-implantation time. The values are given using an independent sample *t*-test

^*^represents statistically significant differences

## Discussion

The increased accuracy of pedicle screws and decreased surgical risks has driven the advent of newer technologies, such as navigation and robotics, over the last two decades [12–15]. These modalities are based on 3D real-time image guidance and thus, have been reported to decrease the incidence of screw malposition. Although studies have been conducted to compare the accuracy and safety of screws with robots or navigation to those with traditional fluoroscopy [1, 16–18], there has been no such comparative study between robotics and navigation.

The first important finding of this study is that the robot is more accurate than navigation for aiding screw implantation. To obtain reliable experimental results, two different methods, screw angle deviation, and the Gertzbein and Robbins scale were used to evaluate the screw position.

Regarding screw angle deviation, to the best of our knowledge, we pioneered this evaluation method for assessing the deviation between the actual and designed screw positions. The accuracy of the robotic and navigational screw placement in a total of six parts C1–7, T1–4, T5–8, T9–12, and L1–S1 was evaluated separately, and it was found that angular deviation existed in both device-assisted screw placements. However, the screw angular deviation in the robotic group was smaller than that in the navigational group in all segments (*p* < 0.05). We analyzed the reason for this result: the robotic group established the screw path by placing the Kirschner needle through the robotic arm, whereas the navigation group manually established the screw channel using an opener under image guidance. In this difference, the robotic arm is much more capable of maintaining a fixed direction in space than the manual arm, so the angular deviation of the robot-assisted screw placement would be closer to the trajectory of the designed screws than the navigation.

Regarding the rate of the acceptable screw (grade A + B), we used the Gertzbein and Robbins scale proposed by Gertzbein et al. to observe the severity of screw penetration through the bone cortex [11]. We found that the incidence of acceptable screws in the robot-assisted group was higher than that in the navigation-assisted group in segments C1–7, and T1–4 (*p* < 0.05), while no significant difference was detected between the two groups in segments T5–8, T9–12, and L1–S1 (*p* > 0.05), indicating that robotic assistance is safer and more valuable than navigation for screw placement in the C1–C4 segment. We analyzed the reasons for this as follows: T5–S1 has a larger mean diameter of the pedicle than C1–C4. Although our angular deviation results showed a slight angular deviation in T5–S1, it was not severe enough to penetrate the bone cortex.

The second important finding of this study was that the robot had a shorter screw placement time than that of the navigation system (*p* < 0.05). We compared the following three time periods: preparation time, screw planning time, and guided screw placement time. No statistical difference was found in screw planning time between the robot and navigation groups (*p* > 0.05). However, the robot had a shorter preparation time and guided-screw-placement time than navigation (*p* < 0.05). We believe that there are two reasons for this result. First, the surgical instruments of the robot do not require manual registration, whereas those of the navigation require activation and calibration. Second, for guided-screw placement, the robotic arm automatically moved to the designed screw trajectory direction after stepping on the foot pedal during robot-assisted screw placement, and the time used was significantly lower than that for navigation-assisted screw placement, when the direction was manually adjusted intra-operatively according to the images.

The third important finding of this study is that a one-time C-arm scan can be used with both robotics and navigation, which is a new technology that has not been previously reported in the literature. The combined use of robotics and navigation has four advantages that benefit the patient. First, simultaneous screw placement can be performed on each side of the spine with the aid of two devices, thereby reducing the operative time. Second, if one of the robots and the navigation do not work properly owing to inaccuracy or hardware failure, screw placement can still be performed with the image guidance of the other device. Third, because both techniques use the same set of image data, the two devices can verify the accuracy of each other intra-operatively. Finally, the simultaneous guidance of screw placement by robotics and navigation does not increase the number of image scans and, therefore, does not increase patient radiation exposure. Therefore, the combined application of robotics and navigation is an optional strategy to improve screw placement accuracy and reduce operative time in patients requiring long-segment screw placement, such as those with scoliosis. In addition, the simultaneous use of both devices is required for the type of hardware used during C-arm image acquisition, which must meet the requirements of using a scale for robotics and a C-arm tracer for navigation to obtain images from only one-time C-arm scans that can be used for both robotics and navigation.

This study has several limitations. First, the combined application of the two devices has some disadvantages, including increased patient hospitalization costs, greater operating room space required, and extended surgical incisions to facilitate the fixation of the robotic and navigated spinal clips to the spinous process. However, the combined application of the two devices provides the benefits of high precision and short operative times in some selected patients, especially those with scoliosis. Therefore, surgeons should thoroughly consider the advantages and disadvantages of combining the two devices and fully communicate them with the patients. Second, although the measurement of the component alignment parameters using post-operative CT images and pre-operative planning images has been well documented, the potential for error still exists between research engineers and investigators. Finally, this study was performed using an experimental model, which can only provide a theoretical basis for clinical applications. Therefore, multicenter, large-scale, randomized controlled studies are needed to further compare the accuracy of robotic and navigation applications in patients.

In conclusion, this controlled study showed that the robot-assisted technique offers better screw placement accuracy and screw placement time than the navigation-assisted technique. In addition, the combined application of robotics and navigation may provide options for some selected patients but with trade-offs.

## Supplementary Information

Below is the link to the electronic supplementary material.

Supplementary file1 (TIF 6889 KB)

Supplementary file2 (TIF 9695 KB)

## Data Availability

Data is not a part of a public repository.
