# Supplementary figures and images for: Robot-assisted versus navigation-assisted screw placement in spinal vertebrae
Source: Int Orthop. 2022 Nov 24;47(2):527–32. doi: 10.1007/s00264-022-05638-0 (PMC9877038; doi:10.1007/s00264-022-05638-0)

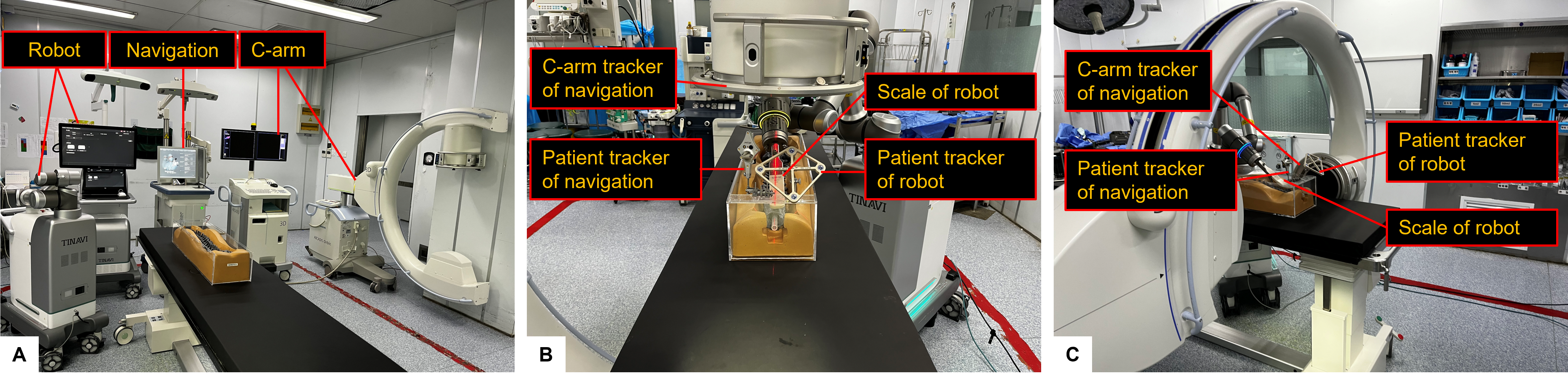

Supplement: Supplementary file 1 — Supplementary file1 (TIF 6889 KB) [file 264_2022_5638_MOESM1_ESM.tif]

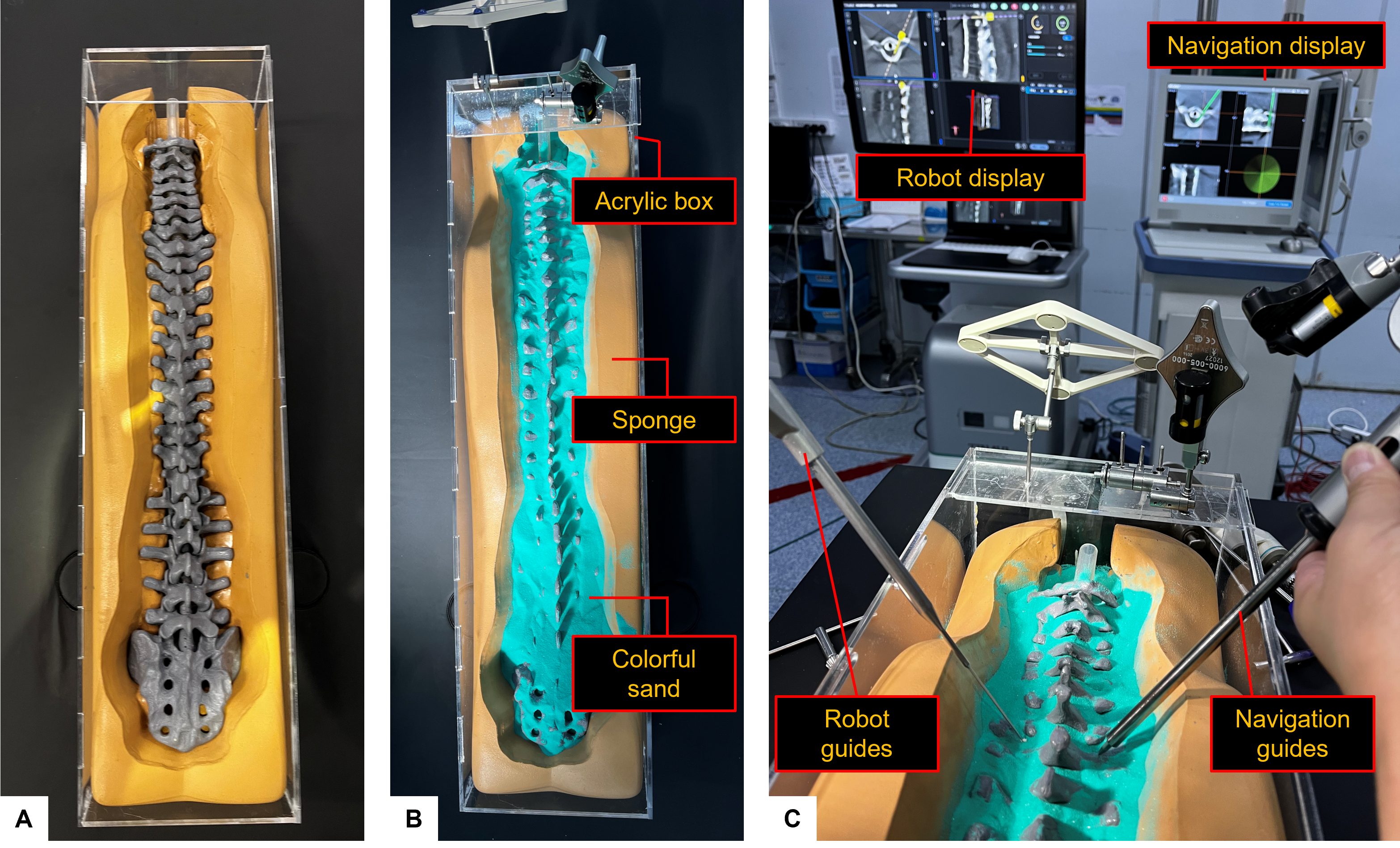

Supplement: Supplementary file 2 — Supplementary file2 (TIF 9695 KB) [file 264_2022_5638_MOESM2_ESM.tif]
